# Dynamics of Bacterial Communities and Their Relationship with Nutrients in a Full-Scale Shrimp Recirculating Aquaculture System in Brackish Water

**DOI:** 10.3390/ani15101400

**Published:** 2025-05-12

**Authors:** Arslan Emmanuel, Yingzhen Wei, Muhammad Naeem Ramzan, Wen Yang, Zhongming Zheng

**Affiliations:** School of Marine Sciences, Ningbo University, Ningbo 315211, China; arslanemmanuel88@gmail.com (A.E.);

**Keywords:** shrimp culture, RAS, bacterial community succession, brackish water, water microbiota, biofilm microbiota

## Abstract

Microbial communities in RASs play a critical role in maintaining water quality and supporting shrimp growth, development, and health. However, their dynamics, particularly in commercial systems, remain poorly understood. This study aimed to improve the understanding of bacterial community dynamics during shrimp culture in RASs. High-throughput amplicon sequencing of the 16S rRNA, PERMANOVA, PCoA, and other statistical analyses were used to investigate the bacterial dynamics. The entire succession process was categorized into three distinct phases, the initial, middle, and final phases, during the shrimp rearing in RASs to elucidate the spatial–temporal dynamics of the bacterial communities. Alpha diversity indicates the evenness of the bacterial community increased in the initial phase, while richness peaked in the middle phase. Notable taxonomic and functional groups within the bacterial community contributed to significant variations in the relative abundance of community composition across these phases. The dominant bacterial phyla in both water and biofilm included Proteobacteria, Actinobacteriota, Bacteroidota, and Patescibacteria. The dominant orders in both environments were Corynebacteriales, Burkholderiales, Rhodobacterales, Flavobacteriales, Saccharimonadales, and Micrococcales. Key bacterial taxa such as *Pseudomonas*, *Mycobacterium*, and *Hydrogenophaga* were critical for microbial community assembly, nutrient cycling, biodegradation, and water quality monitoring. Nitrite, ammonium, and nitrate were positively correlated with *Mycobacterium, Rheinheimera, Taeseokela*, and *Thermomonas*, while negatively correlated with the *Cloacibacterium* community composition. These findings expand our understanding of the underlying mechanisms of bacterial community succession in RASs with intensive rearing of shrimp and suggest that stabilizing environmental variables could be a useful management tool for promoting and maintaining healthy aquaculture environments.

## Simple Summary

Shrimp farming in brackish water faces challenges in maintaining water quality and shrimp health, often due to changes in bacterial communities and nutrient levels. This study explored how bacterial communities shift over time in a full-scale shrimp recirculating aquaculture system (RAS), which reuses water to reduce environmental impact. The goal was to understand how these bacteria interact with nutrients like nitrogen and phosphorus, which are critical for shrimp health but can be harmful in excess. By monitoring water samples throughout a shrimp farming cycle, the researchers found that bacterial communities changed in response to nutrient levels, with certain beneficial bacteria becoming more dominant during stable water conditions. These findings suggest that managing bacterial balance can help control nutrient buildup and improve shrimp health and productivity. This study highlights the importance of understanding microbial dynamics in aquaculture systems, offering insights that can lead to more sustainable and efficient shrimp farming practices. This research benefits society by supporting food security, reducing environmental pollution, and promoting liable aquaculture development.

## 1. Introduction

Recirculating aquaculture systems (RASs) represent advanced aquaculture technologies that have the potential to support the cultivation of a diverse range of seafood species but not limited to prawns, salmon, sea bass, shrimp, trout, and tuna across various environments [1]. These systems use a closed-loop filtration process to optimize water quality (temperature, salinity, pH, oxygen) for species growth [2] while minimizing effluent discharge, such as ammonium, solid waste, and carbon dioxide [3], converted into non-toxic compounds that can be safely reused within the aquaculture system [4]. Previous studies have demonstrated that RASs can reduce water consumption by 90–99% and significantly decrease ammonia concentrations [5]. Additionally, the adoption of RASs has been found to enhance growth performance, survival rates, and overall production efficiency [6].

Bio-filters, which rely on microbial communities for nutrient processing and maintaining water quality, play a critical role in the effectiveness of RASs, making them essential for sustainable shrimp farming practices [7]. Furthermore, the flexibility of RASs in supporting a diverse range of species highlights their potential as a sustainable solution for expanding global seafood production, especially in regions with restricted access to conventional marine environments [8]. Consequently, RASs are often referred to as “hyper-intensive” or “super-intensive” farming [9]. Moreover, it has been involved in the cultivation of *Litopenaeus vannamei*, commonly known as white-leg shrimp, which are economically significant worldwide [10]. By 2018, production of this species had reached 3.5 million tons, establishing it as one of the most crucial species in aquaculture [11]. The *L. vannamei* is particularly favored for its superior disease resistance, rapid growth rates, and high tolerance to stressful conditions [12]. These attributes have contributed to the increasing interest in utilizing RAS technology to optimize its cultivation [13]. This intensive farming approach can generate significant yields, often reaching up to 500 tons of shrimp per hectare annually, all within a relatively small water volume [14].

Microbial communities in RASs have been extensively studied for aquaculture systems [15], but shrimp RAS microbial communities remain rarely explored. Microorganisms play a pivotal role in maintaining the stability of the aquaculture environment within RASs [1]. The bio-filter, a core component of RASs, functions as a microbial system specifically designed to remove nitrogenous waste byproducts generated during the breakdown and oxidation of shrimp proteins [16]. Despite its essential function, the bio-filter remains a complex system with a limited understanding of its underlying mechanisms. The efficiency of the bio-filter is primarily influenced by the composition and abundance of functional microbial communities within it, which directly affect its capacity for water purification [17]. These microbial communities frequently form complex biofilms that play a critical role in biotransformation processes, converting toxic substances into less harmful compounds, thus improving water quality and promoting the health of cultured organisms [18] whereas, denitrifying bacteria can be used in another treatment step to reduce water usage even further by converting nitrate into nitrogen gas that can be removed from the system [19]. Different bacteria in nitrifying biofilters of RASs have been reviewed [20,21,22,23], but the knowledge of temporal dynamics in full-scale RASs is under-reviewed.

The microbial communities in RASs can respond rapidly to changes in the environment, with different selection pressures acting on the microbial communities [24]. The different forces driving these selection pressures include feed and feeding regimes, the make-up water, management routines, system design, physicochemical water quality, and the shrimp itself [25,26]. In addition to the bio-filter, microorganisms present within the culture water itself contribute to environmental stability and support the overall well-being of the cultured species [27]. These microbes directly interact with aquaculture organisms, influencing their health, growth, and productivity [28]. Consequently, a comprehensive understanding of the bacterial community composition and its fluctuations within biofilters and culture water is vital for optimizing the design, operation, and functionality of RASs [29]. Solutions to maintain beneficial microbial communities in RASs, which is important for system management and control, are practically lacking [24,30].

Previous research has shown that during shrimp culture in RASs, different bacterial communities were involved in improving the water quality and the health of shrimp [31] while also focusing on the relationship between bacterioplankton community stability and eutrophication levels [32]. Taking this scenario, in this study, we observed the following: (1) the successional changes in the bacterial community in water during different phases of shrimp culture in RASs; (2) the bacterial community shift in biofilm during shrimp culture in RASs; and (3) the relationship between bacterial communities and environmental factors during shrimp culture in RASs. This study will explore the fundamental mechanism of specific bacterial taxa behind the water quality management during the shrimp growth period, specifically during the last stage of cultivation.

## 2. Materials and Methods

### 2.1. Culture System Setup

The field experiment was conducted at Jufu Agriculture Farm, located in Beilun District, Zhejiang Province, Ningbo, China. Four full-scale RASs on the farm were selected for this study. Each RAS setup consisted of four replicated rearing tanks, each with a volume of 38 m^3^, a water pump, a microfiltration unit, and a bio-filter tank with a volume of 40 m^3^. On 1 May 2023, juvenile *L. vannamei* shrimp were stocked in the rearing tanks at a density of 25,000 individuals per tank, equivalent to approximately 658 individuals per cubic meter. The bio-filter used moving-bed biofilm reactor (MBBR) technology, utilizing polyethylene elastic media submerged in water. The bio-filter was inoculated with rearing water one month in advance to facilitate biofilm formation. All of the RASs were housed within a greenhouse to maintain a relatively stable temperature. The salinity of the rearing water was approximately 5 ppt, and the shrimp were cultured for a duration of three months and harvested on 18 July 2023. Each day, the shrimps were fed three times (at 9:00, 14:00, and 20:00) using commercial pellets (48% proteins; Table A2). The daily feeding rate was 6% of the biomass at the beginning and adjusted according to the actual feeding condition. The total feed, residual feed, and feces, as well as water exchange quantity and nitrogen concentration, were monitored to calculate nitrogen input and output. Furthermore, water temperature and turbidity were measured daily in each system before feeding to assess the culture condition and estimate the effect of bacteria on the nitrogen budget.

### 2.2. Sample Collection and Water Quality Analysis

Sampling was conducted on days 15, 31, 46, 61, and 76 of the experimental period. A total of 80 samples, including both water and biofilm, were collected from four replicate random sampling positions designated within the bio-filters (at 9:00) to monitor succession processes. The shrimp samples’ average wet weight was recorded on both the first day (0.02 ± 0.005 g) and the last day (13.89 ± 2.71 g) of the experiment, i.e., the 90th day (Table A1). Water samples were filtered using 0.45 μm polycarbonate membrane filters and stored at −20 °C until analysis. Microbial water and biofilm samples were filtered using a 0.22 μm membrane filter until the membrane surface became visibly obscured. The filtered membranes were then stored in sterile Eppendorf tubes at −80 °C and transported on dry ice for subsequent DNA extraction and high-throughput sequencing analysis. Prior to analysis, the biofilm samples were immersed in sterile water for 5 min, followed by ultrasonic treatment at a specified temperature (<30 °C) for 3 min using an ultrasonic vibration instrument model XM-3200UVF (Shenzhen Xiangmin Instrument and Equipment Co., Ltd., Shenzhen, China) at 50 Hz. Water quality parameters such as pH, salinity (S), dissolved oxygen (DO), temperature (T), chemical oxygen demand (COD_Mn_), active phosphate (PO_4_^3−^-P), nitrite (NO_2_^−^-N), nitrate (NO_3_^−^-N), ammonia nitrogen (NH_4_^+^-N), total nitrogen (TN), and total phosphorus (TP) were measured on a weekly basis. Temperature, salinity, pH, and dissolved oxygen were measured with a water quality analyzer. Nutrient concentrations, including NH_4_⁺-N, NO₂^−^-N, NO_3_^−^-N, PO_4_^3−^-P, TN, and TP, were determined by SmartChem450 (KPM Analytics, Westborough, MA, USA). The content of NH₄⁺-N was analyzed using hypobromite oxidation, while NO_3_^−^-N was reduced through a zinc–cadmium column. Nitrate concentrations were measured spectrophotometrically using naphthalene ethylenediamine for NO_2_^−^-N detection. The concentration of PO_4_^3−^-P was determined spectrophotometrically using the phosphomolybdenum blue method. TN and TP were analyzed by potassium persulfate oxidation. COD was measured using a modified permanganate method with an alkaline solution, followed by titration with potassium permanganate.

### 2.3. Processing of DNA Sequencing Data

The bacterial DNA was extracted in this study using the Minkgene Water DNA kit (Guangdong Magigene Biotechnology Co., Ltd., Guangzhou, China) for 16S rRNA sequencing. The yield and quality of the extracted DNA were assessed using a NanoDrop One spectrophotometer (Thermo Fisher Scientific, Waltham, MA, USA). Universal primers 338F (forward; 5∲-ACTCCTACGGAGGCAGCA-3∲) and 806R (reverse; 5∲-GGACTACHVGGGTWTCTAAT-3∲) were utilized for PCR amplification of the bacterial genome. The PCR products were sequenced on the Illumina HiSeq 2500 platform (Guangdong Magigene Biotechnology Co., Ltd., located in Guangzhou, China) using paired-end sequencing with a read length of 250 bp. Sequence assembly and analysis were conducted on the raw sequencing data using the USEARCH program, version 11.0.667_I8. The UNOISE3 algorithm parameters unoise_alpha = 2, minsize = 8, and default settings were utilized for denoising, error correction, chimeric sequence removal, and generation of zero-radius operational taxonomic units (ZOTUs). Taxonomic classification of the ZOTUs was performed by aligning them against the SILVA database version SSU Ref NR 99 138.1 to obtain taxonomic information.

### 2.4. Statistical Analysis

Statistical analyses were conducted using R software version 4.3.2. Visualizations were generated with the ‘ggplot2’ package unless otherwise stated. A one-way analysis of variance (ANOVA; *p* < 0.05) was conducted to assess differences among samples with respect to water quality parameters, as well as the α-diversity (*p* < 0.05) indices using SPSS version 20, whereas R version 4.3.2 was used to analyze β-diversity. Additionally, the Adonis function() from the ‘vegan’ package in R (version 4.3.2) was used to perform permutation multivariate analysis of variance (PERMANOVA; *p* < 0.05). This analysis was conducted to examine differences in community composition between groups and to assess the influence of environmental factors on these differences. Principal coordinate analysis (PCoA) [33] was performed using the ‘cmdscale()’ function from the ‘ape’ package, along with the ‘ddply()’ function from the ‘plyr’ package in R version 4.3.2. The analysis aimed to evaluate the bacterial community structure, which was then visualized for interpretation. A stacked histogram of relative abundance percentages was generated using the ‘ggplot2’ package in R version 4.3.2 for effective visualization. A differential analysis cladogram of bacterial genera present in water and biofilms was constructed using linear discriminant analysis effect size (LEfSe) to identify potential biomarkers at the genus level. Heatmap visualization was generated in R version 4.3.2 using the ‘pheatmap’ package for data representation. Redundancy analysis (RDA) was performed to explore the relationship between environmental factors and microbial community structure, utilizing the ‘vegan’ package in R version 4.3.2. Bray–Curtis dissimilarity was calculated at different time points between groups, and statistical differences were assessed using the Kruskal–Wallis test (*p* < 0.05) in R version 4.3.2 [34].

## 3. Results

### 3.1. Temporal Change in Bacterial Communities in Water and Biofilm Environments

The PERMANOVA test was applied to assess the significance of differences between groups. A significant difference was observed among the groups (Table 1; *p* < 0.05). Pairwise comparisons indicated the presence of three significantly distinct groups (initial, middle, and final), suggesting the existence of three successional stages in the bacterial communities of both environments (Table 1; *p* < 0.05).

**Figure 1:**
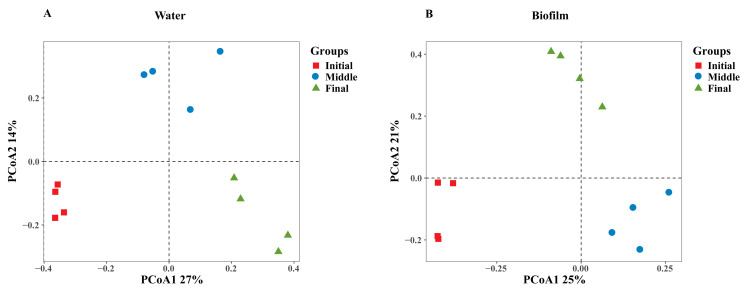
PCoA based on Bray–Curtis’s matrix indicates differences in bacterial community composition in the system. Bacterial communities in water (**A**) and biofilm (**B**). Initial, days 15–31; middle, days 46–61; and final, day 76.

Furthermore, principal coordinate analysis (PCoA) revealed significant temporal effects on the bacterial communities over the rearing period in both the water and biofilm (Figure 1A,B). In the water column, PCoA1 and PCoA2 accounted for 27% and 14% of the total variation (Figure 1A), respectively, while in biofilms, these axes explained 25% and 21% of the variation (Figure 1B).

The diversity indices indicated overall stability in diversity, with notable temporal fluctuations, aligning with three distinct phases: the initial phase (days 15–31), the middle phase (days 46–61), and the final phase (day 76). The *p*-values indicated that biofilm diversity increased significantly over time while water diversity remained stable (Table A4, *p* < 0.05). In the water samples, the Shannon diversity index peaked initially and, thereafter, declined in the middle and final phases. Conversely, the Simpson diversity index was highest in the middle phase compared to the other sampling times. In the biofilm samples, the Shannon diversity index reached its peak in the middle, followed by a significant decrease in subsequent phases. The Simpson diversity index exhibited substantial variation across different time points (Table 2, *p* < 0.05).

### 3.2. Bacterial Community Composition in Water and Biofilms

**Figure 2:**
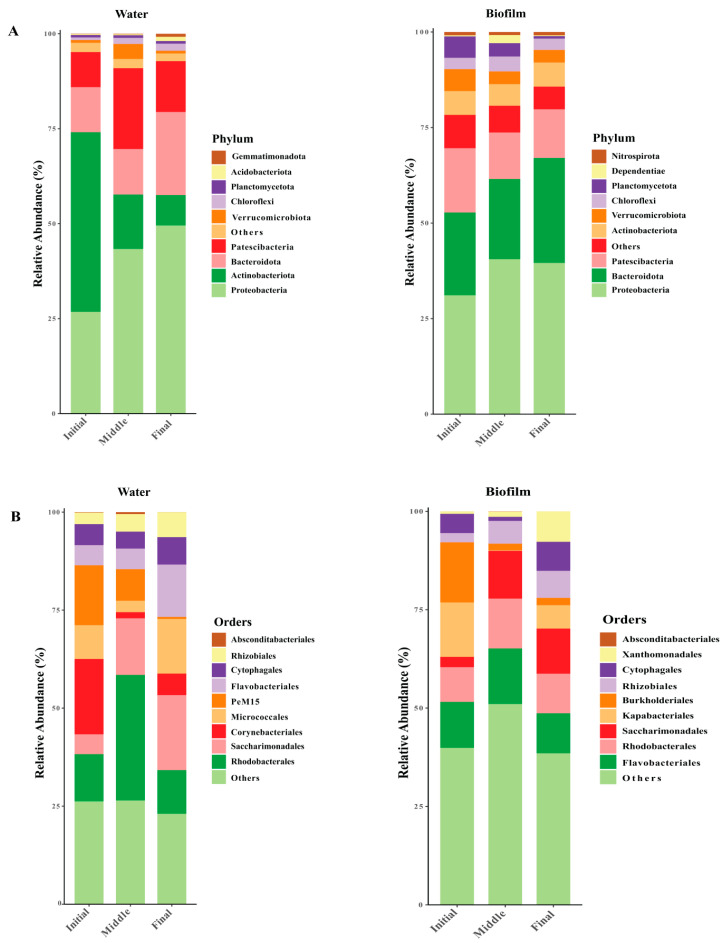
Relative abundance of bacterial communities’ dominant phylum and order levels (top 10) at different times. Bacterial composition in water and biofilm at the phylum level (**A**). Bacterial composition in water and biofilm at the order level (**B**).

A total of 4221 operational taxonomic units (OTUs) were derived from 2,339,952 high-quality sequences collected from 20 samples. Over the course of a 90-day experimental period, significant shifts in the composition of dominant bacterial taxa were observed, emphasizing the dynamic nature of microbial communities. The taxonomic composition of the bacterial community revealed distinct shifts in microbial phyla across the experimental groups. Among the top most abundant phyla, Proteobacteria emerged as the dominant phylum in the final phase, comprising 49.5 ± 0.04% in water, whereas during the middle phase, it accounted for 40.5 ± 0.01% in the biofilm. Furthermore, Bacteroidota was the second predominant group in the final phase, accounting for 21.8 ± 0.12% in the water and 27.4 ± 0.04% in the biofilm. A notable decline in Actinobacteriota, the third most abundant phyla, was observed during the final phase, with its relative abundance of 7.9 ± 0.05% in the water. Meanwhile, in the biofilm, Patescibacteria, categorized as the third most abundant phyla, declined to 12.8 ± 0.06% (Figure 2A). Order-level analysis revealed that Corynebacteriales and Burkholderiales were the most abundant taxa in the initial phase. This was followed by Rhodobacterales and Flavobacteriales, which dominated in the middle phase, whereas, in the final phase, Saccharimonadales and Micrococcales emerged as the most prevalent orders in both water and biofilm (Figure 2B).

### 3.3. Shrimp Growth Performance

The growth performance of shrimp culture in RAS is shown in Table A1. The shrimp achieved an average growth rate of 0.154 g/day, a survival rate of 72.21%, and a feed conversion ratio (FCR) of 1.81 (Table A3).

### 3.4. Relationships Between Microbial Communities and Environmental Factors

**Figure 3:**
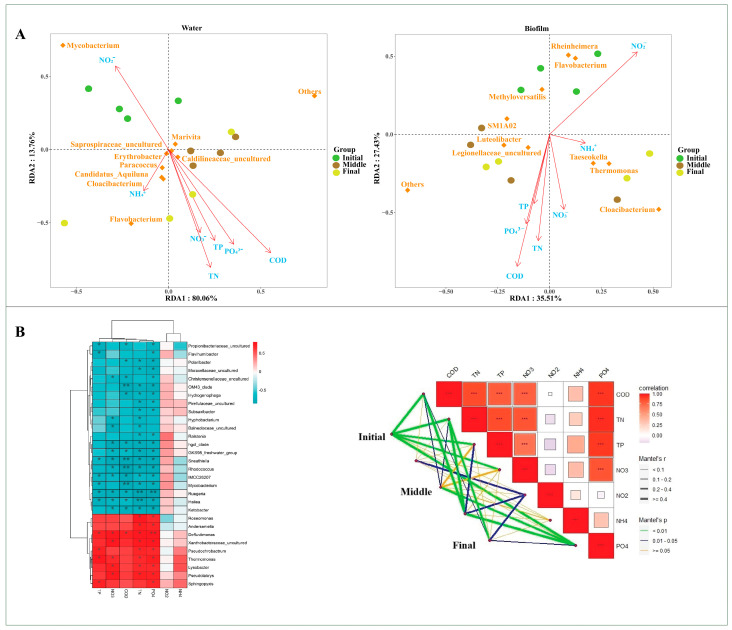
Exploring the relationship between microbial communities and environmental factors using RDA (**A**). RDA explores the relationship between water and biofilm microbial community genera and total nitrogen (TN), chemical oxygen demand (COD_Mn_), nitrite (NO_2_^−^-N), phosphate (PO_4_^3−^-P), total phosphorus (TP), nitrate (NO_3_^−^-N), and ammonia (NH_4_^+^-N). Environmental correlation of microbial communities, as evaluated by a heat map (* *p* < 0.05, ** *p* < 0.01, *** *p* < 0.001) and Mantel tests (**B**).

Redundancy analysis of bacterial communities and nutrient concentrations revealed significant correlations between TP, TN, NO_2_^−^-N, NH_4_^+^, and NO_3_^−^-N. In the water column, *Mycobacterium* exhibited a strong positive correlation with NO_2_^−^-N, whereas *Cloacibacterium* and *Flavobacterium* showed negative correlations with NH_4_^+^ (Figure 3A). In the biofilm samples, *Rheinheimera* and *Flavobacterium* showed a positive correlation with NO_2_^−^-N, while *Taeseokela* and *Thermomonas* were positively correlated with NH_4_^+^. Additionally, *Cloacibacterium* demonstrated a positive correlation with NO_3_^−^-N (Figure 3A). Furthermore, the heat map revealed significant correlations between various environmental factors and the distribution of microorganisms within a RAS (Figure 3B). A Mantel test revealed a strong association between PO₄^3^⁻-P and microbial communities (r > 0.4, *p* < 0.001), emphasizing the influence of phosphorus on microbial community structure. TP, TN, COD_Mn_, NO_3_^−^-N, and NH_4_^+^-N exhibited positive correlations with microbial communities (r > 0.3, *p* < 0.05), whereas NH_4_^+^-N and NO_2_^−^-N displayed negative associations (Figure 3B).

Nutrient analysis demonstrated a consistent increase in water nutrient concentrations throughout the study period: initial (days 15–31), middle (days 46–61), and final (day 76). Bacterial community composition changed significantly over time, whereas different environmental factors had a significant impact on the bacterial communities (Table A5, *p* < 0.05), particularly total nitrogen (TN), total phosphorus (TP), and nitrate (NO₃⁻-N), with significant increases observed from the middle to the final phase. This period indicates nitrogen enrichment and enhanced nitrification. Additionally, organic pollution levels showed an upward trend, as evidenced by the increasing chemical oxygen demand (COD_Mn_) from initial to final. Nitrite (NO_2_^−^-N) concentrations exhibited a complex temporal pattern, peaking initially before decreasing, while ammonia (NH_4_⁺-N) levels fluctuated, with initially high concentrations that decreased in the middle phase before reaching their peak in the final stage. Phosphate (PO_4_^3−^-P) concentrations also increased substantially, particularly from middle to final, correlating with the trend in TP and suggesting potential nutrient enrichment with ecological implications (Table 3, *p* < 0.05).

### 3.5. Identification of Biomarkers

**Figure 4:**
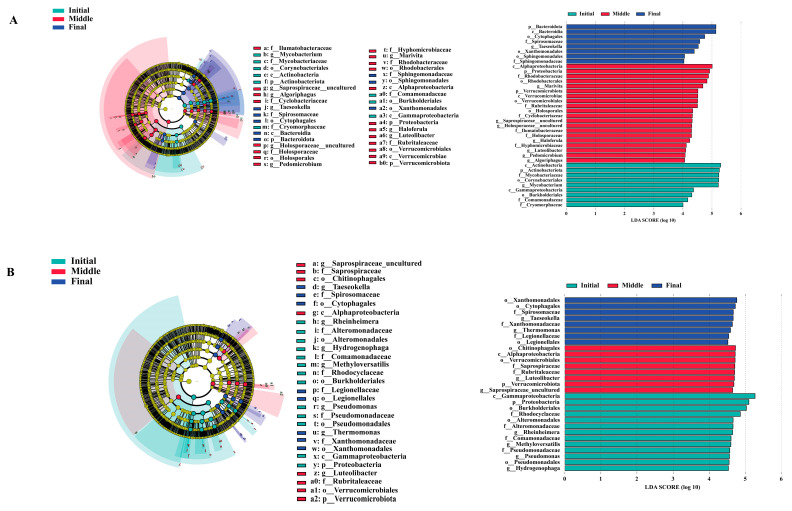
Biomarkers identified during the bacterial community succession process. Water cladogram of bacterial communities and LDA scores with a threshold value of 4 identified the size of differentiation among the three different times (**A**). Biofilm cladogram of bacterial communities and LDA scores with a threshold value of 4.5 identified the size of differentiation among the three different times (**B**).

The LEfSe analysis identified significant potential biomarkers across three experimental groups: initial, middle, and final. A total of seven genera in both water and biofilm samples were identified as potential biomarkers, with an LDA threshold value of 4. In the water column of the initial group, *Mycobacterium* emerged as the key biomarker. In the middle group, *Marivita*, *Haloferula*, *Luteolibacter*, *Pedomicrobium*, and *Algoriphagus* were identified as the dominant biomarkers, while in the final group, *Taeseokella* was identified as the predominant biomarker (Figure 4A). Conversely, in the biofilm community, the initial group was characterized by potential biomarkers such as *Rheinheimera*, *Methyloversatilis*, *Pseudomonas*, and *Hydrogenophaga*. The middle group included *Luteolibacter*, whereas, in the final group, *Taeseokella* and *Thermomonas* were identified as the dominant biomarkers (Figure 4B).

## 4. Discussion

### 4.1. Temporal Dynamics of Microbial Communities

RASs for shrimp culture have gained increasing popularity due to their ability to recycle water, minimize waste discharge, and provide stable environmental conditions for optimal shrimp growth [35]. In RASs, water quality is closely monitored, and biofilm, which forms on surfaces within the system, plays a vital role in the biological filtration process by hosting beneficial microorganisms that break down organic matter and nitrogenous waste [36]. The integration of biofilm into RASs has been shown to enhance water quality, reduce disease risk, and improve shrimp health and growth [37]. The efficiency of biofilm in RASs is crucial for achieving sustainable shrimp farming with minimal environmental impact [38]. In our study, we examined that bacterial communities in both water and biofilm environments of the shrimp RAS undergo distinct successional changes over time. The shifts are closely linked to environmental fluctuations, highlighting the dynamic nature of microbial ecosystems in aquaculture systems as confirmed by PCoA and PERMANOVA analyses (Figure 1, Table 1). This aligns with previous findings, which show that as the environments fluctuate, different bacterial taxa may become more or less competitive, leading to the observed temporal restructuring of the microbial community [39]. This pattern of microbial succession is a well-documented phenomenon wherein communities progressively shift through different stages, often culminating in a more stable community structure as the ecosystem reaches an equilibrium; such changes are indicative of the community’s adaptive responses to dynamic ecological pressures [40]. Previous research supports the significance of temporal dynamics in bacterial communities, particularly in water and biofilm ecosystems [41]. The significant fluctuations in nutrient concentrations, particularly nitrogen and phosphorus, in this study are consistent with the findings of Dmitrijs et al. [42], who observed that nutrient enrichment induces shifts in microbial community structure, often favoring taxa capable of nitrification or organic matter degradation. A comparison of α-diversity indices between water and biofilm environments reveals significant differences in bacterial community dynamics. Notably, biofilms exhibited higher diversity indices (Chao1, Richness, and Shannon) in the initial days (Table 2) compared to water, suggesting that biofilms may provide a more stable and favorable environment for bacterial growth and colonization in the early stages [43]. These findings align with previous research, which has demonstrated that biofilm habitats typically support more diverse microbial communities due to factors such as surface attachment, nutrient gradients, and protection from environmental stresses [44]. However, both environments displayed a decline in diversity at later stages, which may reflect an ecological shift toward fewer, more competitive species that thrive in stable biofilm or water conditions. This reduction in diversity over time is a well-documented phenomenon in microbial community studies, wherein successional processes and competitive exclusion play a significant role in community structuring [45]. Nutrient analysis revealed increasing concentrations of total nitrogen (TN), total phosphorus (TP), and nitrate nitrogen (NO_3_^−^-N) in the water over the study period, which aligns with similar research focused on aquaculture environments [24]. The significant rise in TN levels from the initial to the final phase highlights the potential for progressive nitrogen enrichment (Table 3) and the importance of effective nutrient management strategies in aquaculture systems to mitigate the risks associated with organic pollution and eutrophication [46]. The upward trend in TP levels and phosphate (PO_4_^3−^-P) concentrations (Table 3) aligns with previous observations that indicate nutrient loading can profoundly influence microbial community structure and function [47]. Additionally, the fluctuations in NO_2_^−^-N levels offer insights into the dynamic nature of nutrient cycling within the system (Table 3). This pattern is consistent with findings suggesting that fluctuating nitrite levels are indicative of shifting nitrification rates and microbial community responses to changing nutrient availability [48]. Similarly, the fluctuations in ammonium nitrogen (NH_4_⁺-N) concentrations, which peaked in the final stage, further illustrate the complexity of nutrient interactions and microbial processing. These observations reinforce the intricate relationships between microbial communities and nutrient dynamics in aquaculture systems [49].

This study revealed the bacterial community composition in both water and biofilm column samples to examine the temporal variations and ecological interactions within these microbial communities associated with shrimp cultivation. The results align with the previous findings and provide valuable insights into the shifts in bacterial diversity and composition, enhancing our understanding of the microbial dynamics in RAS systems and their potential implications for shrimp health and system performance [50]. The observed shifts in bacterial community composition align with previous studies examining microbial dynamics in response to environmental perturbations [51]. The dominance of Proteobacteria in the final phase of our experiment likely reflects a response to increased nutrients or other ecological factors that favor the proliferation of this phylum (Figure 2). This aligns with findings from similar studies that highlight the resilience of Proteobacteria to environmental fluctuations such as nutrient availability, temperature fluctuations, oxygen levels [52], nitrogen removal, and water quality management [53]. Many nitrogen-removing bacteria, including ammonia-oxidizing bacteria (AOB) and nitrite-oxidizing bacteria (NOB), belong to the phylum Proteobacteria [54]. The decline in Actinobacteriota in the final phase was particularly noteworthy (Figure 2). This aligns with previous research indicating that Actinobacteria, especially those within the Corynebacteriales order, are sensitive to changes in environmental conditions such as pH and organic matter availability [55]. The observed rise in Bacteroidota, which was associated with the degradation of complex organic compounds and an increase in oxygen availability, supports the notion that the final phase of our study was marked by shifts in resource utilization and microbial metabolism [56]. At the order level, Corynebacteriales (*Phylum Actinobacteria*) and *Burkholderiales* (*Phylum Pseudomonadota*) were the most abundant taxa in the initial phase (Figure 2B), which could suggest a shift in the microbial community from early colonizers to more stable, mature communities as the environmental conditions over time [57]. The dominance of *Rhodobacterales* and *Flavobacteriales* in the middle phase (Figure 2B) of this study is consistent with previous research that has recognized these taxa as significant players in biofilm maturation and organic matter degradation [58] facilitating COD removal [59]. The members of the order *Saccharimonadales* (*Phylum Patescibacteria*) and *Micrococcales* (*Phylum Actinobacteria*) demonstrated a significant increase in the final phase (Figure 2B), which is consistent with studies that have identified members of this order as key contributors to biofilm formation and organic matter cycling in aquatic environments [60]. This suggests that *Saccharimonadales* and *Micrococcales* may play a significant role in biofilm development and stabilization under the experimental conditions. The rise in unclassified taxa in the later phases of the experiment also supports findings from microbial diversity studies, which often report the rise in low-abundance taxa as environmental conditions change [61]. This phenomenon may reflect microbial niche specialization or the adaptation of previously rare organisms to new ecological opportunities [62].

### 4.2. Association of Bacterial Community with Physiochemical Parameters

The interaction between microorganisms and environmental variables is essential for optimizing aquaculture practices and enhancing ecosystem health [63,64]. During this study, RDA analysis revealed that environmental factors significantly influence microbial community composition (Figure 3A,B). NH_4_^+^-N and NO_2_^−^-N exhibited distinct correlations with microbial communities (Figure 3A), suggesting that these factors influence community structure in unique ways. The positive correlation between *Mycobacterium* and nitrite (NO_2_^−^-N) in the water column aligns with previous research suggesting that certain bacteria are specialized in utilizing nitrogen compounds during the nitrogen cycle [65]. Similarly, *Cloacibacterium* and *Flavobacterium* exhibited negative correlations (Figure 3A) with ammonia (NH_4_⁺-N), indicating a preference for environments with lower levels of this compound, in line with observations by Chun et al. [66], where these genera were associated with organic matter degradation in low-nutrient environments. The positive correlations between *Rheinheimera* and *Flavobacterium* with NO₂⁻-N in biofilms (Figure 3A) further support their roles in nitrogen cycling, as these genera contribute to organic matter degradation and the nitrogen cycle [67,68]. The positive correlations of *Taeseokela* and *Thermomonas* with NH_4_⁺-N (Figure 3A) suggest that these genera potentially participate in ammonium oxidation or utilization, consistent with research linking them to nitrogen cycling in biofilm environments [69].

### 4.3. Identification of Biomarkers and Function Analysis

LEfSe analysis results in our study provided critical insights into the temporal dynamics and bacterial succession within the microbial communities of the RAS. In the initial stage of the water column, *Mycobacterium* emerged as a dominant biomarker (Figure 4A). Previous research has highlighted that this genus is known for its resilience in oligotrophic conditions and its potential roles in biodegradation, making it a prominent taxon in aquatic systems [70]. During the mid-successional phase, *Marivita*, *Haloferula*, *Luteolibacter*, *Pedomicrobium*, and *Algoriphagus* were identified as the dominant biomarkers (Figure 4A). Previous findings indicate that genera *Marivita* and *Haloferula*, both belonging to the Alphaproteobacteria, are associated with organic matter degradation, nutrient cycling, improved water quality, and enhanced bioremediation potential [71]. Additionally, *Luteolibacter* enhanced microbial mineralization, whereas *Pedomicrobium*, known for biofilm formation and metal oxidation [72], likely contributed to the development of beneficial biofilms and trace metal regulation in the RAS [73]. Furthermore, the presence of *Algoriphagus*, adept at degrading complex polysaccharides, suggests a role in organic matter breakdown, promoting system cleanliness and reducing pathogenic risks [74,75]. In the final stage, *Taeseokella* was identified as the predominant biomarker (Figure 4A). Previous research indicated that though relatively less studied, this genus has been reported in marine and aquaculture environments, with potential roles in nutrient cycling and antimicrobial compound production [76]. Its dominance in later stages suggests the establishment of a mature and stable microbial community capable of sustaining homeostasis and resisting pathogenic invasions [77]. In the biofilm column, the initial stage was characterized by *Rheinheimera*, *Methyloversatilis*, and *Pseudomonas* as key biomarkers (Figure 4B). Previous research indicated that *Rheinheimera* is noted for its antimicrobial properties and potential as a biological control agent [78], while *Pseudomonas* is a well-documented biofilm former in aquatic systems [79]. *Methyloversatilis* plays a pivotal role in carbon and nitrogen cycling, facilitating detoxification during early biofilm development [80], whereas *Hydrogenophaga* is known for its hydrogen oxidation and nitrate reduction capabilities. Its activity under low-oxygen conditions supports nitrogen removal, contributing to reduced total ammonia nitrogen (TAN) and nitrite concentrations [81,82]. In the mid-successional stage, the concurrent presence of *Luteolibacter* reflects increased metabolic diversity and biofilm complexity, promoting organic matter degradation and reducing the risk of eutrophication [83,84]. In the final stage, *Taeseokella* and *Thermomonas* dominated the biofilm communities (Figure 4B). Previous research indicated that these genera are linked to organic matter degradation, stress tolerance, and thermo-tolerance, which are critical for long-term biofilm stability under fluctuating environmental conditions [85]. Their enrichment suggests a functionally stable and resilient microbial consortium that enhances nitrification and denitrification processes, improves nutrient recycling, and supports overall system stability and shrimp health while minimizing the need for external interventions [86].

## 5. Conclusions

This study emphasizes the dynamic nature of bacterial communities within brackish water RASs during shrimp culture, highlighting their close association with temporal changes in environmental parameters. Successional shifts in microbial composition were strongly influenced by nutrient concentrations, particularly phosphorus and nitrogen, which emerged as key environmental drivers. The identification of distinct bacterial taxa, such as *Mycobacterium*, *Flavobacterium*, and *Rheinheimera*, linked to nutrient cycling, organic matter degradation, and biofilm formation, highlights their ecological significance within RAS environments. Our results revealed clear phase-specific microbial assemblages and biomarkers reflecting the influence of environmental gradients on microbial succession. These findings contribute valuable insights into microbial community dynamics and their functional roles in nutrient processing and ecological stability. Future research should focus on elucidating the specific metabolic functions of key microbial taxa to better inform microbial management strategies and enhance the sustainability and performance of RAS-based shrimp aquaculture systems.

## Figures and Tables

**Table 1:** Pairwise differences between bacterial communities in the water column and biofilm at different times—PERMANOVA analysis based on adonis function (* *p* < 0.05, ** *p* < 0.01, *** *p* < 0.001).

	Water		Biofilm	
Time Point	R^2^	*p*	R^2^	*p*
Overall (PERMANOVA)	0.247	0.001 ***	0.311	0.001 ***
Initial (days 15–31)	0.207	0.002 **	0.241	0.002 **
Middle (days 46–61)	0.278	0.002 **	0.394	0.037 *
Final (day 76)	0.137	0.001 ***	0.187	0.001 ***

**Table 2:** α-diversity of bacterial communities (mean ± SD) in water and biofilm. Different letters specify the significant difference between groups (days) at the same column and same medium (water or biofilm) (*p* < 0.05, one-way ANOVA; Tukey’s HSD).

Medium	Time	Chao1	Richness	Shannon	Simpson
Water	Initial	2642.3 ± 186.5	2194.9 ± 152.6	6.93 ± 0.47	0.04 ± 0.02 ^a^
	Middle	2599.7 ± 303.7	2118.5 ± 311.2	6.24 ± 1.01	0.07 ± 0.05 ^b^
	Final	2481.9 ± 124.6	1994 ± 161.3	6.17 ± 0.56	0.05 ± 0.01 ^ab^
Biofilm	Initial	2807.3 ± 151.2	2363.3 ± 150.7	7.59 ± 0.61	0.03 ± 0.02 ^b^
	Middle	2818.4 ± 158.7	2343.6 ± 142.5	7.66 ± 0.35	0.02 ± 0.01 ^a^
	Final	2511.8 ± 269.9	2012 ± 229.5	7.09 ± 0.20	0.03 ± 0.004 ^ab^

**Table 3:** Nutrient content of water at different times (mean ± SD, mg/L). Different letters specify the significant difference between groups at the same row (*p* < 0.05, one-way ANOVA; Tukey’s HSD).

Parameters (mg/L)	Day 15	Day 31	Day 46	Day 61	Day 76
COD_Mn_	11.88 ± 2.54 ^b^	11.34 ± 2.06 ^b^	21.10 ± 1.84 ^a^	21.32 ± 2.73 ^a^	23.00 ± 5.71 ^a^
TN	16.88 ± 2.93 ^c^	18.97 ± 1.09 ^c^	37.50 ± 5.54 ^b^	40.83 ± 19.32 ^b^	72.96 ± 13.80 ^a^
TP	9.48 ± 7.68 ^b^	7.43 ± 4.86 ^b^	8.94 ± 3.18 ^b^	11.45 ± 6.25 ^b^	41.61 ± 26.39 ^a^
NO_3_^−^-N	4.46 ± 0.59 ^d^	7.63 ± 5.24 ^c^	4.51 ± 1.53 ^d^	14.17 ± 3.05 ^b^	30.08 ± 2.07 ^a^
NO_2_^−^-N	1.78 ± 0.25 ^a^	0.22 ± 0.07 ^c^	0.31 ± 0.22 ^bc^	0.44 ± 0.12 ^b^	0.31 ± 0.04 ^bc^
NH_4_^+^-N	0.79 ± 0.40 ^b^	0.44 ± 0.62 ^bc^	0.12 ± 0.10 ^c^	0.09 ± 0.11 ^c^	0.91 ± 0.16 ^a^
PO_4_^3−^-P	1.00 ± 0.37 ^d^	1.00 ± 0.12 ^d^	3.17 ± 1.05 ^c^	5.58 ± 1.02 ^b^	8.59 ± 1.65 ^a^

Note: total nitrogen (TN), total phosphorus (TP), chemical oxygen demand (COD_Mn_), ammonia nitrogen (NH_4_^+^-N), nitrate (NO_3_^−^-N), nitrite (NO_2_^−^-N), and orthophosphate (PO_4_^3−^-P).

## Data Availability

The raw data supporting the conclusions of this article will be made available by the authors on request.

## Appendix A

**Table A1:** Growth performance of shrimp in RAS.

Initial					Final			
	Stocking Density (ind m^−3^)	Weight (g)	Number of Shrimps per Tank	Total Weight (kg) per Tank	Stocking Density (kg m^−3^)	Weight (g)	Number of Shrimps per Tank	Total Weight (kg) per Tank
RAS	658 ± 0.0032	0.02 ± 0.005	25,000	0.5 ± 0.125	9.14 ± 1.53	13.89 ± 2.71	18,053	250.7 ± 48.92

**Table A2:** The percentages of shrimp feed ingredients over cultivation.

Ingredients	Postlarvae (Length < 3 cm)	Juveniles (Length 3~6 cm)	Adults (Length > 6 cm)
Crude protein	48.0	43.0	43.0
Crude fat	6.0	6.0	6.0
Crude fibre	4.0	6.0	6.0
Crude ash	17.0	18.0	18.0
Total phosphate	1.4	1.0	1.0
Lysine	3.2	2.3	2.4
water	10.0	12.0	12.0

**Table A3:** Shrimp growth performance indicators.

Parameters	Value	Remarks
Daily Growth Rate	0.154 g	Per day
Survival Rate	72.21%	At harvesting
Specific Growth Rate (SGR)	7.27%	Per day based on log weight change
Initial Biomass	0.5 kg	Per tank
Final Biomass	250.7 kg	Per tank
Weight Gain (Biomass)	250.2 kg	Per tank
Total Feed Given	452.16 kg	Estimated over 90 days
Feed Conversion Ratio (FCR)	1.81	Feed used per kg weight gain
Protein in Feed	48%	Commercial pellet composition
Protein Intake	216.93 kg	48% of total feed
Protein Efficiency Ratio (PER)	1.15	Weight gain per kg protein intake

**Table A4:** α-diversity of bacterial communities (mean ± SD) in water and biofilm. Significant differences between groups (days) within the same column are indicated (* *p* < 0.05, ** *p* < 0.01, one-way ANOVA; Tukey’s HSD).

		Chao1	Richness	Shannon	Simpson
*p*-value	Water	0.8	0.7	0.3	0.22
	Biofilm	0.06	0.01 **	0.04 *	0.12

**Table A5:** Quantitative effects of sampling time and environmental factors on variation in bacterial community structure using one-way ANOVA (*p* < 0.05).

	Days		Factors		Days × Factors	
	F	*p*	F	*p*	F	*p*
Community composition	109.4	0.000	180.2	0.000	18.6	0.000
